# *IL6* genetic perturbation mimicking IL-6 inhibition is associated with lower cardiometabolic risk

**DOI:** 10.1038/s44161-025-00700-7

**Published:** 2025-08-26

**Authors:** Lanyue Zhang, Murad Omarov, Lingling Xu, Emil deGoma, Pradeep Natarajan, Marios K. Georgakis

**Affiliations:** 1 Institute for Stroke and Dementia Research, LMU University Hospital, LMU Munich, Munich, Germany; 2 Tourmaline Bio Inc., New York, NY USA; 3 Cardiovascular Research Center and Center for Genomic Medicine, Massachusetts General Hospital, Boston, MA USA; 4 Cardiovascular Disease Initiative, Broad Institute of Harvard and MIT, Cambridge, MA USA; 5 Department of Medicine, Harvard Medical School, Boston, MA USA; 6 Program in Medical and Population Genetics and Cardiovascular Disease Initiative, Broad Institute of MIT and Harvard, Cambridge, MA USA; 7 Munich Cluster for Systems Neurology (SyNergy), Munich, Germany

**Keywords:** Genetics research, Drug development

## Abstract

Human genetics supports a causal involvement of IL-6 signaling in atherosclerotic cardiovascular disease, prompting the clinical development of anti-IL-6 therapies. Genetic evidence has historically focused on *IL6R* missense variants, but emerging cardiovascular treatments target IL-6, not its receptor, questioning the translatability of genetic findings. Here we develop a genetic instrument for IL-6 signaling downregulation comprising *IL6* locus variants that mimic the effects of the anti-IL-6 antibody ziltivekimab and use it to predict the effects of IL-6 inhibition on cardiometabolic and safety endpoints. Similar to *IL6R*, we found that genetically downregulated IL-6 signaling via *IL6* perturbation is associated with lower lifetime risks of coronary artery disease, peripheral artery disease and ischemic atherosclerotic stroke in individuals of European and East Asian ancestry. Unlike *IL6R* missense variants linked to bacterial infections, the *IL6* instrument was associated with lower risk of pneumonia hospitalization. Our data suggest that IL-6 inhibition can reduce cardiovascular risk without major unexpected safety concerns.

## Abstract (web summary)

Zhang et al. show that genetically simulated IL-6 inhibition is associated with a reduced risk of cardiovascular disease and no increase in infection risk, supporting the use of emerging pharmacological treatments targeting IL-6 rather than its receptor.

## Main

Atherosclerotic cardiovascular disease (ASCVD) remains the leading cause of death worldwide^1^, with major projected increases in its burden^2,3^. Inflammation has emerged as a new therapeutic target, with colchicine being the first anti-inflammatory drug to be approved for lowering ASCVD risk^4,5^. A new generation of anti-inflammatory treatments is advancing through clinical development^6,7^. Agents targeting interleukin-6 (IL-6) signaling have attracted interest due to converging evidence implicating a role in atherosclerosis^8^. Two anti-IL-6 monoclonal antibodies are actively tested in phase 3 trials (ziltivekimab^9^ in ZEUS^10^ and clazakizumab in POSIBIL_6_ESKD^11^) and one in a phase 2 trial (pacibekitug in TRANQUILITY)^12^. Human genetic studies have been integral in supporting the causal involvement of IL-6 signaling in ASCVD and contributed to the emergence of these development programs. Genetic variants near the gene coding for the IL-6 receptor (IL-6R) that reduce signaling activity have been associated with lower lifetime risks of coronary artery disease (CAD), ischemic stroke (IS) and peripheral artery disease (PAD)^13–21^. However, evidence has been limited to *IL6R* missense variants owing to their high frequency in European populations and their strong effects on downstream signaling. As emerging ASCVD therapeutics exclusively target IL-6, not IL-6R, this gap in evidence raises concerns about the translatability of genetic effects to pharmacological IL-6 inhibition.

The complexity of IL-6 signaling could imply differences in targeting IL-6 versus IL-6R. IL-6 signals through its membrane-bound receptor on leukocytes and hepatocytes (classic signaling) or via its soluble receptor on other cell types (trans-signaling)^22,23^. While IL-6 blockade could inhibit both pathways, IL-6R inhibitors may exert differential effects on classic and trans-signaling depending on their affinity for membrane-bound versus soluble IL-6R^22,23^. Moreover, IL-6R targeting can induce compensatory increases in circulating IL-6 levels, which is not observed with IL-6-targeting^24^. A recently described mode of cluster signaling that involves trans-presentation of the IL-6/membrane-bound IL-6R complex from dendritic cells to gp130 on T cells, priming pathogenic T_H_17 responses^25^, further highlights the complexity of IL-6 signaling. This pathway may be inhibited by IL-6R inhibitors but not anti-IL-6 antibodies^22,25^. Differences might also be related to the safety profile of these approaches. While secondary analyses of the CANTOS trial that targeted the upstream regulator of IL-6 signaling, IL-1β, showed a reduction in cardiovascular risk^26,27^, there was also a higher risk of fatal infections^28^, meriting further investigation for optimal targeting approaches. Exploring whether genetic perturbation in the gene coding for IL-6 is associated with ASCVD and acceptable safety outcomes could inform investment and development decisions as results from pivotal trials targeting IL-6 are awaited^10^.

Here we developed a genetic proxy (instrument) of IL-6 signaling downregulation, leveraging variants in the locus of the gene encoding IL-6, and used it to predict the effects of pharmacological IL-6 inhibition on cardiometabolic and safety endpoints. We validated the instrument by showing (1) consistent effects with the anti-IL-6 antibody ziltivekimab across eight biomarkers and (2) significant effects on autoimmune outcomes, for which IL-6 signaling inhibition was efficacious in previous trials. Similar to *IL6R*, we found genetically downregulated IL-6 signaling via *IL6* perturbation to be associated with lower lifetime risks of CAD, PAD and atherosclerotic stroke in individuals of European and East Asian ancestry. Furthermore, the *IL6* instrument showed associations with lower risk of type 2 diabetes and increases in high-density lipoprotein (HDL) particles. IL-6 signaling downregulation via *IL6* perturbation was associated with a lower risk of pneumonia hospitalization, in contrast to associations of *IL6R* missense variants with a higher risk of bacterial infections. Phenome-wide analyses replicated these findings and supported repurposing opportunities for IL-6 inhibition toward depression and gallstone disease. On the safety end, we found warning signals for migraine, open-angle glaucoma and pregnancy-related maternal hemorrhage.

## Results

### Study overview

**Fig. 1:**
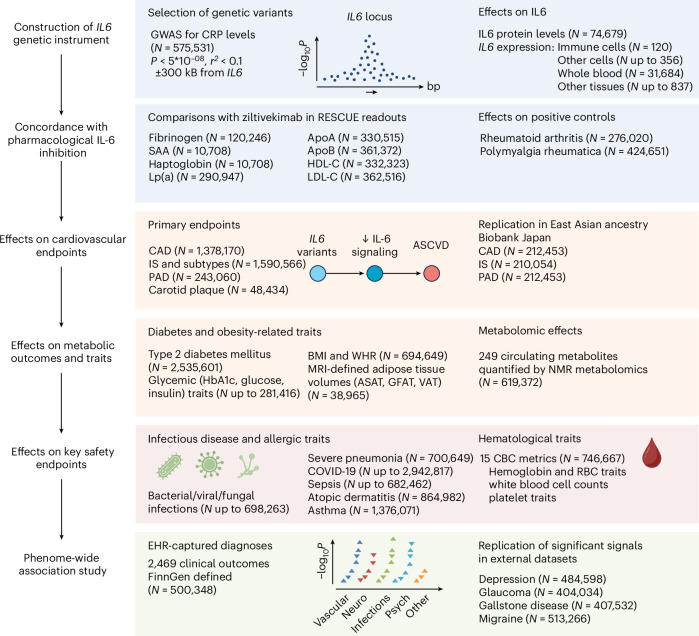
Overview of the study design. The steps of the analytical approach and the samples used. We developed a genetic instrument of IL-6 signaling downregulation consisting of CRP-lowering variants in *IL6*, demonstrated concordance between its biomarker profile and those observed with IL-6–blocking therapeutics and tested associations with atherosclerotic cardiovascular endpoints, metabolic traits, infectious disease and allergic outcomes and hematological traits, as well as electronic health record (EHR) diagnoses in a PheWAS study. Neuro, neurological; Psych, psychiatric.

Our study design is summarized in Fig. 1. We searched for genetic variants in the locus of the gene encoding IL-6 (*IL6*, chromosome 7p15.3) that are associated with downregulation of IL-6 signaling, serving as proxies for inhibition of the pathway. As a readout, we used serum C-reactive protein (CRP) levels, a well-established biomarker of IL-6 signaling activity^29,30^ that is used to test pharmacological inhibition of IL-6 signaling in clinical trials^9,31^. Focusing on an area spanning 300 kilobases (kb) upstream and downstream of *IL6*, we screened for independent variants (clumped at *r*^*2*^ < 0.1) that were associated with CRP levels (*P* < 5 × 10^−8^) in a genome-wide association study (GWAS) of 575,531 European individuals^32^. Variants meeting our selection criteria were combined into a genetic instrument, which was validated against clinical trials testing IL-6 signaling inhibitors. Subsequently, we performed two-sample drug target Mendelian randomization (MR)^33^ for ASCVD outcomes (primary endpoints), metabolic traits and key safety endpoints (Fig. 1). The datasets used in our analyses are described in Supplementary Table 1.

### *IL6* genetic instrument regulates *IL6* expression in immune cells, reducing circulating CRP and IL-6

**Fig. 2:**
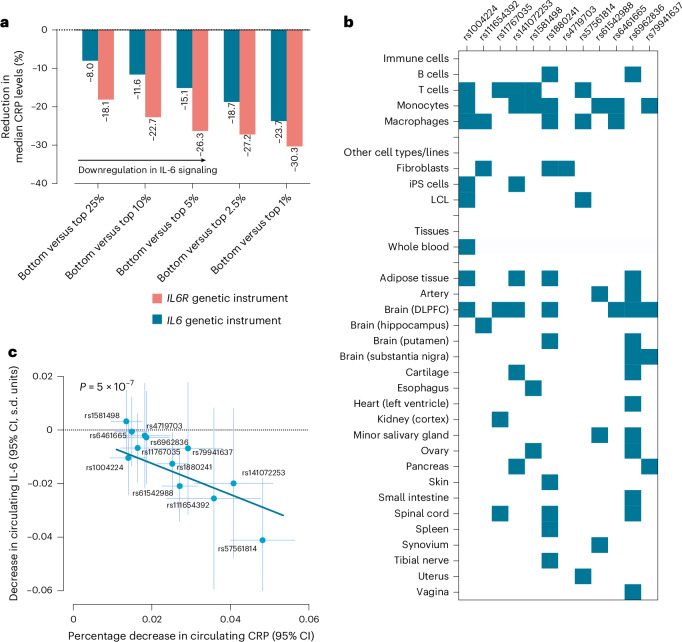
The developed *IL6* genetic instrument is associated with measurable reductions in serum CRP mainly by influencing *IL6* expression and circulating IL-6 levels. **a**, The effects of the 12-variant *IL6* genetic score versus a previously described 26-variant *IL6R* genetic score^15,17,20^ on reductions in CRP levels across percentiles. The bars represent the percentage reduction in median CRP levels across different genetic score percentiles. **b**, The effect estimates of genetic variants comprising the *IL6* instrument on *IL6* expression across immune cells, other cell types or lines and tissues, as extracted from the eQTL catalog resource^35^—blue boxes indicate nominally significant (two-sided *P* < 0.05) effects on expression. **c**, The association of genetically proxied downregulation of IL-6 signaling through the *IL6* 12-variant, as captured by decreases in serum log-transformed CRP (*x* axis) with circulating IL-6 levels (scaled in s.d. units, *y* axis) on IVW MR analysis. The blue line shows the regression slope with the overall association (two-sided *P* = 5 × 10^−7^). Each point represents the effect estimates for a genetic variant, with horizontal and vertical error bars indicating the 95% CIs for CRP and IL-6 reductions, respectively. CRP associations are derived from the UK Biobank (*N* = 464,264), IL-6 associations are derived from ref. ^36^ (*N* = 74,679). DLPFC, dorsolateral prefrontal cortex; iPS cells, induced pluripotent stem cells; LCL, lymphoblastoid cell line. Source data

Our main genetic instrument consisted of 12 single-nucleotide polymorphisms (SNPs) in a region spanning the *IL6* gene and 300 kb upstream or downstream. To avoid confounding effects via neighboring genes^34^, we developed two alternative instruments consisting of eight and three SNPs selected from regions spanning 100 kb and 10 kb upstream and downstream of *IL6*, respectively. The SNPs included in each instrument are listed in Supplementary Table 2. The *F* statistics of the included SNPs ranged from 35 to 183, suggesting sufficient instrument strength for downstream analyses. When combined, the 12-, 8- and 3-variant instruments explained 0.17%, 0.13% and 0.07% of the variance in serum CRP, respectively. In individual-level data in the UK Biobank (*N* = 464,264, mean age ± s.d. of 57.1 ± 8.1 years, 54.2% female; study characteristics in Supplementary Table 3), we found strong associations of our instruments, analyzed as genetic scores, with CRP levels. Individuals in the bottom versus top percentile of the main instrument score distribution had a difference of 24% in median CRP levels (Fig. 2a). A similar discrimination in CRP levels was also observed for the eight-variant score, whereas individuals in the bottom versus top fifth percentile of the distribution of the three-variant genetic risk scores (GRS) showed a median difference of 11.8% in CRP (Supplementary Table 4). To ensure our analyses reflect the natural variation captured by the instruments and avoid extrapolation beyond their range, we scaled their effects to correspond to a 24% decrease in CRP levels.

**Extended Data Fig. 1:**
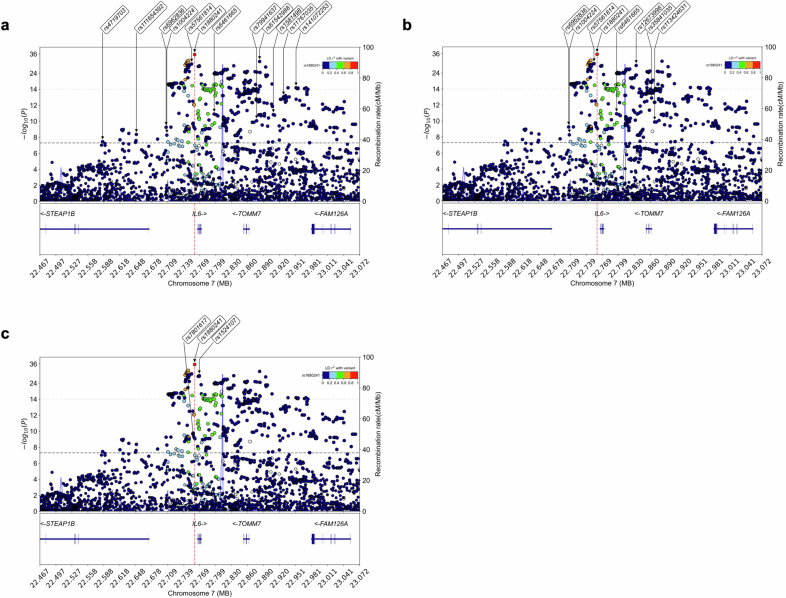
Associations of genetic variants in the *IL6* locus (300 kb upstream and downstream of *IL6*) with serum CRP levels. Annotated are the variants selected for the (**a**) 12-variant (300 kb upstream or downstream to *IL6*), (**b**) 8-variant (100 kb upstream or downstream to *IL6*), and (**c**) 3-variant (10 kb upstream or downstream to *IL6*) genetic instrument of IL-6 signaling downregulation, as used for the current analyses. The data are derived from the genome-wide association study meta-analysis performed by Said et al. (Nat Commun 13, 2198; 2022). Plots were generated using the GWASLab Python package^81^. Abbreviations: kb, kilobases; CRP, Creactive protein; IL-6, interleukin-6; LD, linkage disequilibrium.

Exploring the biological effects of the selected variants, none of them was located within *IL6-*coding regions (Supplementary Table 2 and Extended Data Fig. 1). To explore potential regulatory effects on *IL6* expression, we assessed whether the SNPs are expression quantitative trait loci (eQTLs) for *IL6* in immune cells, other cell lines, whole blood and other tissues using the eQTL catalog resource^35^. Interestingly, we found evidence for all 12 genetic variants included in the main instrument to be influencing *IL6* mRNA expression in specific cells or tissues (Fig. 2b and Supplementary Table 5). There was evidence of enrichment of eQTLs for immune cells, particularly monocytes and macrophages, where all but three genetic variants showed significant effects on *IL6* expression. Similarly, there was evidence that all SNPs in the eight- and three-variant instruments were eQTLs for *IL6* in one or more cell types or tissues (Supplementary Table 5).

**Extended Data Fig. 2:**
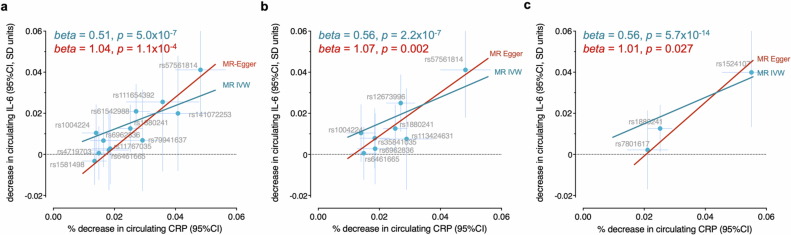
Associations of genetically proxied IL-6 signaling activity through (a) 12, (b) 8, and (c) 3 *IL6* genetic variants, as captured by decreases in serum log-transformed CRP (x-axis) with circulating IL-6 levels (scaled in SD units, y-axis) in inverse-variance weighted and Egger Mendelian randomization analysis. Each point represents an effect estimate for a genetic variant, with horizontal and vertical error bars indicating the 95% confidence intervals (CI) for CRP and IL-6 reductions, respectively. CRP associations derived from UK Biobank (n = 464,264 individuals); IL-6 associations derived from Konieczny et al. (Commun Biol 8, 34, 2025; n = 74,679 individuals). All reported p-values are two-sided. Abbreviations: CRP, C-reactive protein; IL-6, interleukin-6; CI, confidence interval; OR, odds ratio; SD, standard deviation; IVW, inverse-variance weighted; MR, Mendelian randomization. Source data

Investigating whether our instruments influence IL-6 protein levels (*N* = 74,679 individuals of European ancestry^36^), we found genetically downregulated IL-6 signaling activity through any of the three *IL6* instruments to be associated with lower circulating IL-6 (decrease of 0.14 s.d. units per 24% decrease in CRP, 95% confidence interval (CI) −0.18 to −0.09; Fig. 2c). While MR-Egger regression showed evidence of directional pleiotropy, the corrected effect estimates were still significant and larger in magnitude than those from the main inverse variance-weighted (IVW) analyses (Extended Data Fig. 2 and Supplementary Table 6). Similarly, we found an association with lower IL-6 levels in cerebrospinal fluid (Supplementary Table 6). As opposed to genetic proxies of IL-6R inhibition^17,19^, our *IL6* genetic instruments had no influence on soluble IL-6R levels (Supplementary Table 6).

### IL6 genetic instrument proxies pharmacological IL-6 inhibition

**Fig. 3:**
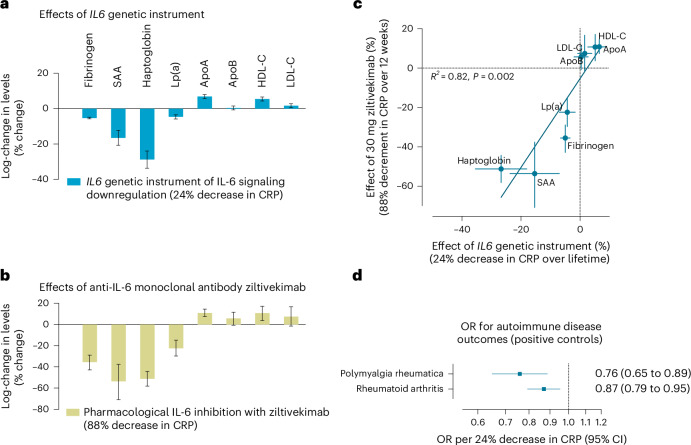
The effects of the *IL6* genetic instrument mirror the effects of IL-6 inhibition as captured in clinical trials. **a**, IVW MR associations of genetically proxied downregulation of IL-6 signaling through perturbation in *IL6* (12-variant instrument, scaled to a 24% decrease in CRP levels) with 8 circulating biomarkers measured in the RESCUE trial^9^. Data are presented as log changes (% change) in levels and error bars correspond to 95% CIs. The *IL6* genetic instrument derived was from a CRP GWAS (UK Biobank + CHARGE; *n* = 575,531). Biomarker associations derived from multiple GWAS datasets as detailed in Supplementary Table 1 (sample sizes vary by biomarkers). **b**, The effects of 30 mg of ziltivekimab (anti-IL-6 monoclonal antibody) administered subcutaneously every 4 weeks over 12 weeks versus placebo on the 12-week change in 8 circulating biomarkers in the RESCUE trial (led to an 88% placebo-adjusted 12-week decrease in serum CRP)^9^. Data are presented as log changes (% change) in levels; error bars correspond to 95% CIs. RESCUE trial: *n* = 66 per group (30 mg ziltivekimab versus placebo). **c**, Correlation of the effects of the IL6 genetic instrument (scaled to 24% decrease in CRP; *n* = 575,531) and ziltivekimab in RESCUE (30 mg ziltivekimab versus placebo; *n* = 66 per group) on the 8 biomarkers (Spearman’s *ρ* = 0.91). Each point represents an effect estimate for a biomarker, as derived by IVW MR with horizontal and vertical error bars indicating the 95% CIs for genetic instrument and ziltivekimab effects, respectively. **d**, IVW MR associations between genetically downregulated IL-6 signaling through perturbation in *IL6* (scaled to a 24% decrease in CRP; *n* = 575,531) and autoimmune disease outcomes in publicly available GWASs (8,156 cases and 416,495 controls for polymyalgia rheumatica and 35,871 cases and 240,149 controls for rheumatoid arthritis). Data are presented as ORs per 24% decrease in CRP and error bars correspond to 95% CIs. All reported *P* values are two sided. Source data

**Extended Data Fig. 3:**
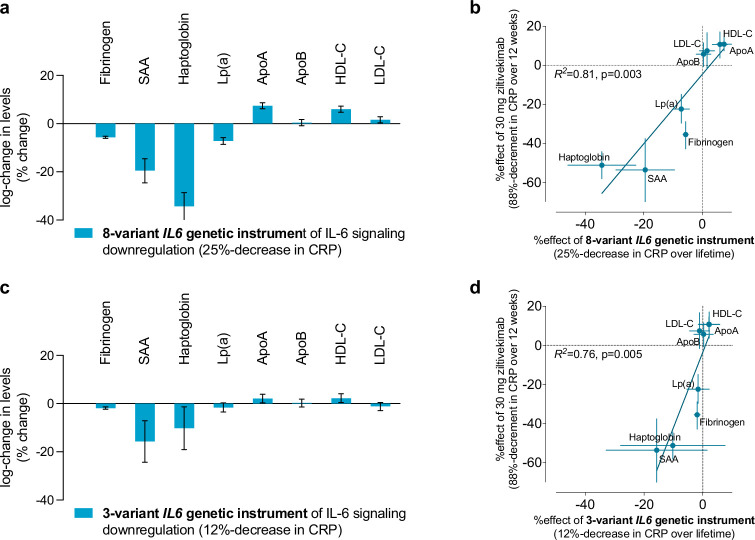
Genetic validation of IL-6 signaling inhibition effects using Mendelian randomization compared with ziltivekimab treatment in the RESCUE trial. Inverse-variance weighted Mendelian randomization associations of genetically proxied downregulation of IL-6 signaling through perturbation in *IL6* using the (**a**) 8- and (**b**) 3-variant instruments (scaled to 25% and 12% decrease in CRP levels, respectively) with 8 circulating biomarkers measured in RESCUE trial. Data are presented as logchanges (% change) in levels; error bars correspond to 95% confidence intervals. *IL6* genetic instrument derived from CRP GWAS (UK Biobank + CHARGE; n = 575,531), Biomarker associations derived from multiple GWAS datasets as detailed in Supplementary Table 1 (sample sizes vary by biomarker). RESCUE effects from n = 66 per group (30 mg ziltivekimab versus placebo). Correlations of the effects of the (**c**) 8- and (**d**) 3-variant *IL6* genetic instrument (CRP GWAS, UK Biobank+CHARGE; n = 575,531) and ziltivekimab in RESCUE (30 mg ziltivekimab versus placebo; n = 66 per group) on the 8 biomarkers (Spearman’s ρ = 0.90 and 0.87, respectively). Each point represents an effect estimate for a biomarker, as derived by inverse-variance weighted Mendelian randomization with horizontal and vertical error bars indicating the 95% confidence intervals (CI) for genetic instrument and ziltivekimab effects, respectively. All reported p-values are two-sided. Abbreviations: CRP, C-reactive protein; IL-6, interleukin-6; CI, confidence interval; OR, odds ratio; LDL-C, low-density lipoprotein cholesterol; HDL-C, high-density lipoprotein cholesterol; SAA, serum amyloid A; Lp(a), lipoprotein(a); ApoB, apolipoprotein B; ApoA, apolipoprotein A; GWAS, genome-wide association study; CHARGE, Cohorts for Heart and Aging Research in Genomic Epidemiology. Source data

The phase 2 trial RESCUE showed that IL-6 inhibition with ziltivekimab leads to changes in fibrinogen, serum amyloid A (SAA), haptoglobin, lipoprotein(a) (Lp(a)), apolipoprotein A (ApoA) and high-density lipoprotein cholesterol (HDL-C) levels^9^. In line with these results, we found that genetically proxied IL-6 signaling downregulation through *IL6* perturbation (12-variant instrument) was significantly associated with lower levels of fibrinogen, SAA, haptoglobin and Lp(a), and higher levels of ApoA and HDL-C across different GWAS datasets (Fig. 3a,b). Remarkably, there was strong consistency (*r*^*2*^ = 0.82, *P* = 0.002) between the effects of 30 mg ziltivekimab over 12 weeks (88% CRP reduction relative to placebo) and those of genetically proxied IL-6 signaling downregulation (scaled at 24% reduction in CRP; Fig. 3c). The results for all biomarkers were robust in sensitivity MR analyses (Supplementary Table 6), as well as when using the eight- and three-variant instruments (Extended Data Fig. 3 and Supplementary Table 7). There was no evidence of directional pleiotropy on MR-Egger analyses.

**Extended Data Fig. 4:**
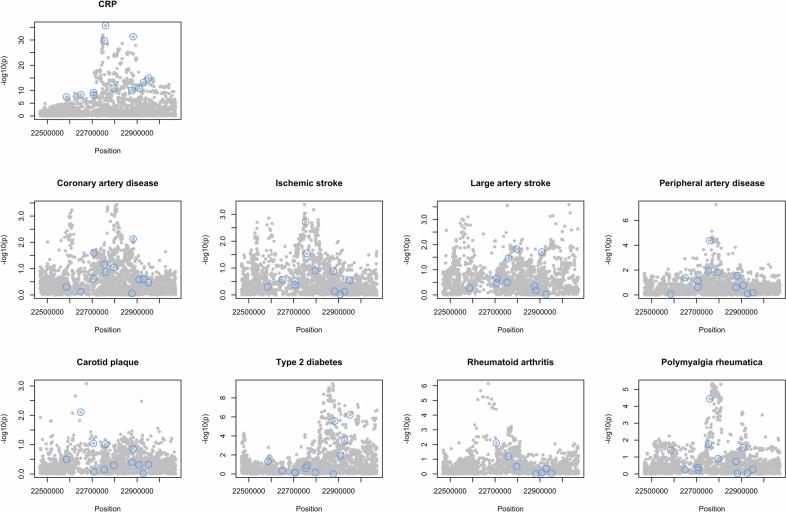
*IL6* locus zoom plots for associations of genetic variants with CRP levels and 8 autoimmune and cardiovascular outcomes that were significant in Mendelian randomization analyses. Blue circles point to genetic variants included in the main 12-variant *IL6* instrument of interleukin-6 signaling downregulation. All reported p-values are two-sided. Abbreviations: CRP, C-reactive protein.

To further validate our genetic instrument against clinical outcomes, we tested, as positive controls, associations with polymyalgia rheumatica and rheumatoid arthritis, for which pharmacological IL-6 signaling inhibition has been proven efficacious in phase 3 trials^37,38^. Similar to *IL6R* perturbation^39,40^, IL-6 signaling downregulation through genetic perturbation in *IL6* was associated with a lower risk of both polymyalgia rheumatica (odds ratio (OR) per 24% decrease in CRP of 0.76, 95% CI 0.65 to 0.89) and rheumatoid arthritis (OR of 0.87, 95% CI 0.79 to 0.95; Fig. 3d). Similar results were obtained with the alternative instruments (Supplementary Table 8), as well as in sensitivity MR analyses (Supplementary Table 9). Colocalization analysis also revealed significant evidence of a shared causal variant between polymyalgia rheumatica and CRP levels in the *IL6* locus (PP.H4 = 0.85; Supplementary Table 10 and Extended Data Fig. 4). These results largely support the validity of the developed genetic instrument as a proxy of IL-6 antagonism.

### *IL6* perturbation is associated with atherosclerotic cardiovascular risk

**Fig. 4:**
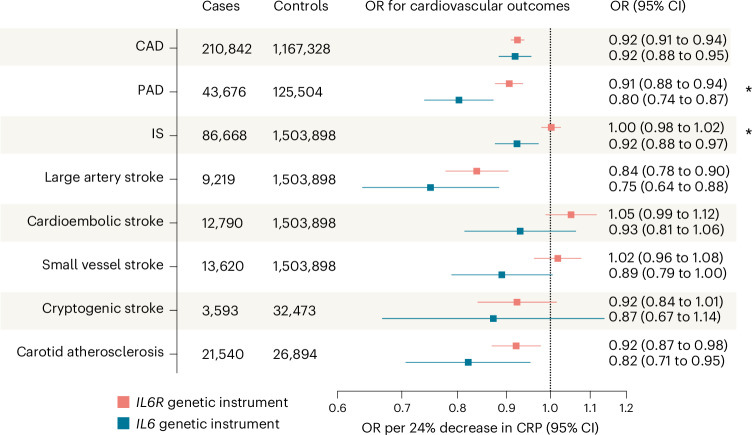
Genetically downregulated IL-6 signaling via perturbation in *IL6* is associated with lower risk of ASCVD. The results from two-sample MR analyses (IVW method) scaled to a 24% decrease in CRP levels. The datasets used for each outcome are detailed in Supplementary Table 1. Data are presented as ORs per 24% decrease in CRP and error bars correspond to 95% CI. Asterisks indicate significant heterogeneity (Cochran’s *Q* test, *P* < 0.05) between the effects of *IL6* and *IL6R* instruments. The exact *P* values and FDR-adjusted *P* values (Benjamini–Hochberg method) are presented in Supplementary Table 9. All reported *P* values are two sided.

**Extended Data Fig. 5:**
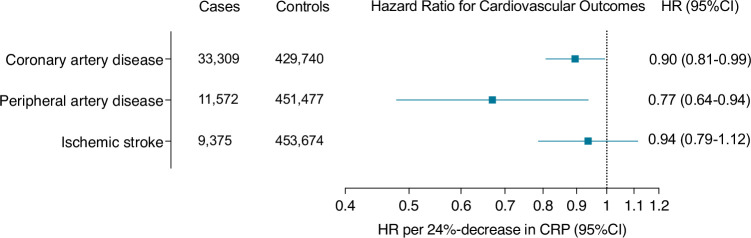
Genetically downregulated IL-6 signaling via perturbation in *IL6* is associated with lower risk of atherosclerotic cardiovascular disease in UK Biobank. Data are presented as hazard ratios per 24%-decrease in CRP; error bars correspond to 95% confidence intervals. Pvalues for coronary artery disease, peripheral artery disease, and ischemic stroke were: p = 0.03, p = 0.009, and p = 0.44, respectively. All reported p-values are two-sided. Abbreviations: IL-6, interleukin-6; CRP, C-reactive protein; CI, confidence interval; HR, hazard ratio.

After establishing the validity of the instrument, we tested associations with ASCVD outcomes using available GWAS datasets (Fig. 4). Genetically downregulated IL-6 signaling through *IL6* perturbation was associated with lower odds of CAD (OR per 24% decrease in CRP of 0.92, 95% CI 0.88 to 0.95), PAD (OR of 0.80, 95% CI 0.74 to 0.87), IS (OR of 0.92, 95% CI 0.88 to 0.97) mainly driven by the large artery atherosclerotic subtype (OR of 0.75, 95% CI 0.64 to 0.88) and ultrasound-defined carotid plaque (OR of 0.82, 95% CI 0.71 to 0.95). A similar effect was found for the alternative carotid atherosclerosis readout of intima media thickness (*β* (mm) of −0.009, 95% CI −0.015 to −0.002; Supplementary Table 6). The findings were highly consistent with the alternative eight- and three-variant instruments (Supplementary Table 8). No evidence of heterogeneity was detected in the main MR analyses (*P* from Cochran’s *Q* >0.05), nor was there evidence of directional pleiotropy (*P* from Egger regression intercept >0.1). The results remained consistent across sensitivity MR analyses (Supplementary Table 9). Comparisons with a previously developed 26-variant *IL6R* instrument of IL-6 signaling downregulation^15,17,20^ revealed directionally consistent results, although there was evidence of larger effects of *IL6* perturbation on odds of PAD and IS (*P* for heterogeneity <0.05; Fig. 4). The results for CAD and PAD were also consistent in one-sample MR analyses in the UK Biobank (Extended Data Fig. 5).

The findings for PAD were further supported by a result indicative of colocalization (PP.H4 = 0.52). While there was no significant evidence of a shared causal variant in the *IL6* locus in colocalization analyses for the remaining ASCVD outcomes, there was also no evidence of distinct causal variants (PP.H3 < 0.2 for all other outcomes) that would suggest bias due to pleiotropic effects of variants in linkage disequilibrium (LD) with the variants comprising our instrument (Supplementary Table 10 and Extended Data Fig. 4). This probably reflects the relatively weak associations of individual variants in the *IL6* locus with ASCVD outcomes, as compared with CRP. This interpretation is supported by the lack of significant heterogeneity in the Heterogeneity in Dependent Instruments (HEIDI) test^41^, which is designed to detect signals arising from LD between independent causal variants (Supplementary Table 10). Although classical colocalization methods assume the presence of a single causal variant, fine-mapping with SharePro identified a single credible set of variants at the locus for CRP levels and all ASCVD outcomes (Supplementary Table 10), providing no evidence of violation of this assumption.

### The cardiovascular effects of genetic *IL6* perturbation are replicated in an East Asian population

**Extended Data Fig. 6:**
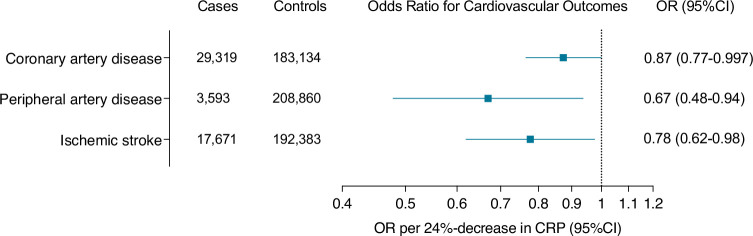
Genetically downregulated IL-6 signaling via perturbation in *IL6* is associated with lower risk of atherosclerotic cardiovascular disease in Biobank Japan. Data are presented as odds ratios per 24% decrease in CRP; error bars correspond to 95% confidence intervals. Results from two-sample Mendelian randomization analyses (inverse variance weighted method) using variant weights and association estimates from Biobank Japan. The exact p-values and FDR-adjusted p-values are presented in Supplementary Table 12. All reported p-values are two-sided. Abbreviations: IL-6, interleukin-6; CRP, C-reactive protein; CI, confidence interval; OR, odds ratio.

As the datasets used in our analyses were largely from European-ancestry individuals (Supplementary Table 1), we performed a cross-ancestry replication in an East Asian population. Using data from Biobank Japan, we found 9 of the 12 variants of our main genetic instrument to be present in the Japanese population and 6 of them remained following clumping with an East Asian LD reference panel (Supplementary Table 11). To weigh the instruments, we used summary statistics for serum CRP levels from Biobank Japan (*N* = 75,391)^42^, and then tested associations with CAD (29,319 cases and 183,134 controls), PAD (3,593 cases and 208,860 controls) and IS (17,671 cases and 192,383 controls)^43^. Genetically proxied IL-6 signaling downregulation via *IL6* perturbation was associated with lower risk of all outcomes (OR for CAD per 24% decrease in CRP of 0.87, 95% CI 0.76 to 0.997; OR for PAD of 0.67, 95% CI 0.48 to 0.94 and OR for IS of 0.78, 95% CI 0.62 to 0.98; Extended Data Fig. 6 and Supplementary Table 12). As not all of the variants influencing CRP were replicated in the East Asian population, we repeated the analysis based on variants achieving an *F* statistic >10 or variants showing a *P* value of <10^−4^. The results were consistent with the main analyses. Furthermore, there was no evidence of heterogeneity or a significant intercept in the MR-Egger analysis (Supplementary Table 12).

### Perturbation in *IL6* is associated with lower diabetes risk

**Fig. 5:**
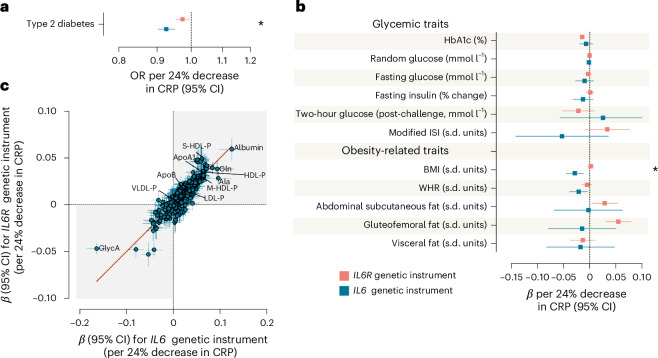
IL-6 signaling downregulation via genetic perturbation in *IL6* is associated with metabolic effects. **a**, IVW MR associations of genetically downregulated IL-6 signaling through perturbation in *IL6* and *IL6R* (scaled to a 24% decrease in CRP) with type 2 diabetes (428,452 cases and 2,107,149 controls). Data are presented as ORs per 24% decrease in CRP and error bars correspond to 95% CIs. The exact *P* values and FDR-adjusted *P* values (Benjamini–Hochberg method) are presented in Supplementary Table 9. **b**, Glycemic and obesity-related traits (the datasets used for each outcome are detailed in Supplementary Table 1). Data are presented as effect estimates per 24% decrease in CRP and error bars correspond to 95% CIs. The exact *P* values and FDR-adjusted *P* values are presented in Supplementary Table 6. **c**, The correlation between the MR effects (IVW method) of genetically proxied IL-6 signaling downregulation through perturbation in *IL6* and *IL6R* (scaled to a 24% decrease in CRP) on NMR metabolomic traits. The exact *P* values and FDR-adjusted *P* values are presented in Supplementary Table 13. Each point represents an effect estimate for a metabolomic trait, with horizontal and vertical error bars indicating the 95% CIs for *IL6* and *IL6R* effects, respectively. All reported *P* values are two sided. Asterisks indicate significant heterogeneity (Cochran’s *Q* test *P* < 0.05) between the effects of *IL6* and *IL6R* genetic instruments. Ala, alanine; Gln, glutamine; HDL-P, high-density lipoprotein particles; LDL-P, low-density lipoprotein particles; M-HDL-P, medium HDL particles; S-HDL-P, small HDL particles; VLDL-P, very low-density lipoprotein particles. Source data

In subsequent analyses, we explored the extent to which genetically proxied IL-6 signaling activity is associated with the risk of type 2 diabetes, as well as other glycemic and obesity-related traits. We found that genetically downregulated IL-6 signaling through either *IL6* or *IL6R* is associated with lower odds of type 2 diabetes, although the effect of *IL6* perturbation was larger in magnitude (OR per 24% decrease in CRP of 0.92, 95% CI 0.90 to 0.94 for *IL6* versus 0.97, 95% CI 0.95 to 0.99 for *IL6R*; Fig. 5a). Although there was evidence of heterogeneity across the 12 variants, the results were consistent in analyses using alternative MR methods (Supplementary Table 9). Exploring glycemic traits, we found no significant effects of *IL6* perturbation on glycated hemoglobin (HbA1c), random fasting or 2-h post-challenge glucose levels, fasting insulin levels or the modified insulin sensitivity index (ISI) (Fig. 5b). However, *IL6* perturbation was associated with lower body mass index (BMI) and a lower waist-to-hip ratio (WHR) (Fig. 5b), with consistent results on sensitivity MR analyses (Supplementary Table 6). We found no significant effects on magnetic resonance imaging (MRI)-quantified metrics of abdominal, subcutaneous, gluteofemoral or visceral fat (Fig. 5b).

Given the previous evidence for an effect of pharmacological IL-6R inhibition on total cholesterol levels^44,45^, we explored the full metabolomic spectrum of the effects of genetic downregulation of IL-6 signaling through perturbation in *IL6* and *IL6R* using GWAS data for 249 metabolites quantified with nuclear magnetic resonance (NMR). The detailed results of this analysis are provided in Supplementary Table 13. In agreement with results for clinically used biomarkers (Fig. 3), there were stronger effects of genetically downregulated IL-6 signaling on the concentration of HDL particles, as captured by cholesterol concentration in different-size HDL particles, HDL particle number and apolipoprotein A1 (ApoA1) levels, when compared with the effects on apolipoprotein B (ApoB)-containing particles, such as low-density lipoprotein (LDL), intermediate-density lipoprotein or very low-density lipoprotein (Fig. 5c and Supplementary Table 13). There were significant associations with higher triglyceride content, especially in HDL particles, in line with previously reported associations of ziltivekimab with higher triglyceride levels^46^. The results were highly consistent for *IL6* versus *IL6R* perturbation (*r*^*2*^ = 0.82). Beyond lipoprotein traits, we found perturbation in both *IL6* and *IL6R* to be associated with higher albumin levels and lower levels of glycoprotein acetyls (GlycA), an NMR biomarker of systemic inflammation^47,48^.

### Genetic IL-6 downregulation linked to lower risks of severe pneumonia, sepsis and COVID-19

**Fig. 6:**
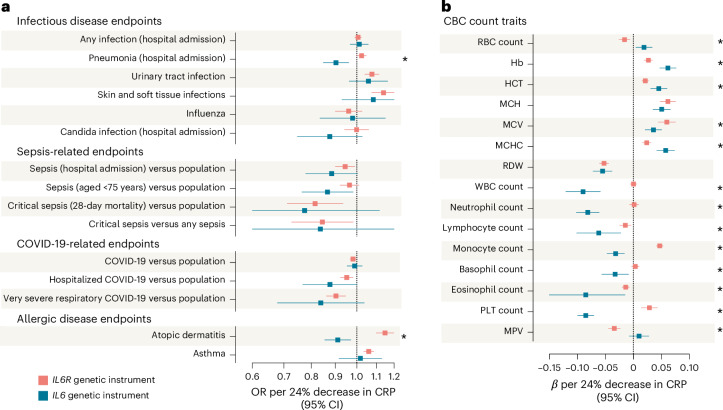
Effects of genetically downregulated IL-6 signaling via perturbation in *IL6* on infection risk, sepsis and COVID-19 outcomes, allergic disease and hematological traits. **a**, IVW MR associations of genetically downregulated IL-6 signaling through perturbation in *IL6* and *IL6R* (scaled to a 24% decrease in CRP) with clinical endpoints related to infectious disease, sepsis, COVID-19 and allergic conditions. Data are presented as ORs per 24% decrease in CRP and error bars correspond to 95% CIs. The exact *P* values and FDR-adjusted *P* values (Benjamini–Hochberg method) are presented in Supplementary Table 9. **b**, CBC count traits. All datasets used for each outcome are detailed in Supplementary Table 1. The exact *P* values and FDR-adjusted *P* values (Benjamini–Hochberg method) are presented in Supplementary Table 6. Data are presented as effect estimates per 24% decrease in CRP and error bars correspond to 95% CIs. All reported *P* values are two sided. Asterisks indicate significant heterogeneity (Cochran’s *Q* test *P* < 0.05) in the effects of *IL6* and *IL6R* genetic instruments. MCHC, mean corpuscular hemoglobin concentration; MPV, mean platelet volume. Source data

Given the risk of immunosuppression with treatments targeting IL-6R^49,50^, we next explored associations with key infectious disease endpoints and complete blood count (CBC) traits (Fig. 6). There was no association with a composite outcome of hospital admission due to any infection (Fig. 6a). Interestingly however, *IL6*-mediated genetic downregulation of IL-6 signaling was associated with lower risk of hospital admission due to pneumonia, in contrast to a higher risk linked to *IL6R* perturbation. On other key bacterial infection endpoints, such as skin/soft tissue and urinary tract infections, we found trends toward a higher risk (similar to *IL6R*), although the effects were not statistically significant. For nonbacterial infections, there was no evidence for higher risk of influenza or candida infections (Fig. 6a). Similar to *IL6R*, we found genetic IL6 downregulation to be associated with lower risk of hospital admission due to sepsis and a sepsis diagnosis in individuals <75 years, as well as hospitalization due to COVID-19. As we previously found an association of an *IL6R* instrument with atopic dermatitis and asthma^16^, we also performed analyses for these endpoints. Notably, perturbation in *IL6* was associated with lower odds of atopic dermatitis and no elevation in odds of asthma (Fig. 6a). The results were consistent on sensitivity analyses (Supplementary Table 9).

Analyses for CBC traits revealed significant effects on red blood cell (RBC), white blood cell (WBC) and platelet (PLT) traits (Fig. 6b). Specifically, genetic IL-6 signaling downregulation through *IL6* was associated with higher RBC count, hemoglobin concentration (Hb), hematocrit (HCT), mean corpuscular hemoglobin (MCH), mean corpuscular volume (MCV) and lower RBC distribution width (RDW). On the other hand, we found associations with lower counts of all WBC subtypes (neutrophils, lymphocytes, monocytes, basophils and eosinophils), which were larger in magnitude than the respective ones for *IL6R* perturbation, as well as lower PLT counts. *IL6* and *IL6R* perturbation had opposite effects on monocyte and PLT counts. The sensitivity analyses are presented in Supplementary Table 6.

### Phenome-wide analyses point to repurposing opportunities and potential safety signals

**Fig. 7:**
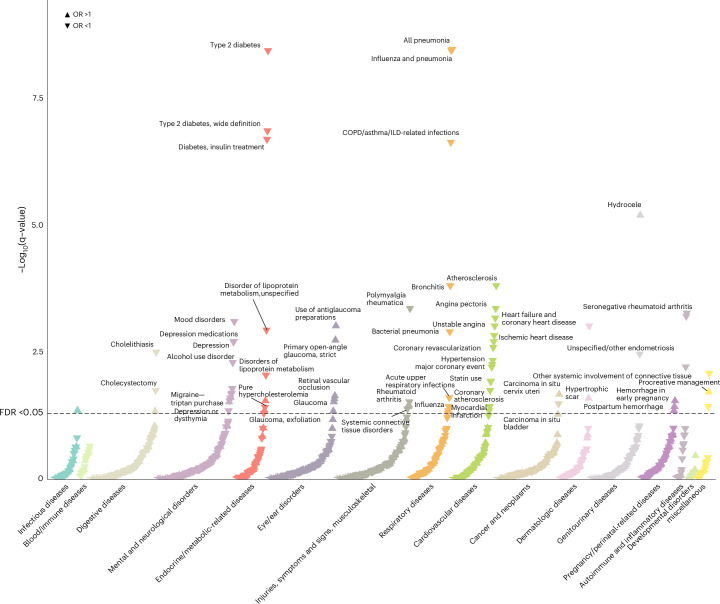
PheWAS study for genetically downregulated IL-6 signaling via *IL6* perturbation highlight repurposing opportunities and safety signals. The results represent association estimates derived from IVW MR analyses for 1,285 binary clinical outcomes with more than 1,000 cases in the population-based FinnGen cohort. The downward arrows represent associations of genetically downregulated IL-6 signaling with lower odds of clinical outcome, whereas the upward arrows represent associations with higher odds of clinical outcome. Annotated are only the clinical outcomes meeting the significance criteria for robustness on sensitivity analyses, as described in the text. The dotted line corresponds to an FDR-corrected *P* value of 0.05. The *y* axis corresponds to the negative 10-base log of FDR-corrected *P* values, as derived from IVW MR analyses. ILD, interstitial lung disease.

As a final step, we conducted a phenome-wide association (PheWAS) using data from the population-based FinnGen study (*N* = 500,348 individuals of Finnish ancestry)^51^. We analyzed 1,285 binary clinical outcomes with more than 1,000 cases. After correcting for multiple comparisons (false discovery rate (FDR)-corrected *P* < 0.05) and rigorously excluding outcomes that lacked consistency across sensitivity MR methods, we found 66 significant outcomes (Supplementary Tables 14 and 15). These signals highlight potential repurposing opportunities or safety concerns (Fig. 7). Genetically downregulated IL-6 signaling via *IL6* perturbation was associated with a lower risk of the following groups of clinical outcomes: (1) atherosclerosis-related cardiovascular endpoints (for example, coronary atherosclerosis, myocardial infarction, angina pectoris and unstable angina, heart failure due to CAD, extracerebral and coronary atherosclerosis), (2) dyslipidemia (for example, statin use, hypercholesterolemia and disorders of lipoprotein metabolism), (3) hypertension, (4) diabetes mellitus, (5) autoimmune connective tissue disorders (for example, rheumatoid arthritis and polymyalgia rheumatica), (6) respiratory infections (for example, bacterial pneumonia, influenza, chronic obstructive pulmonary disease (COPD)-related infections and bronchitis), (7) gallstone disease (cholelithiasis and cholecystectomy), (8) depression-spectrum outcomes, (9) alcohol dependence and use disorder and (10) miscellaneous outcomes (endometriosis, in situ bladder carcinoma and cerebral cysts). On the safety end, genetically downregulated IL-6 signaling via *IL6* perturbation was associated with a higher risk of the following outcomes: (1) glaucoma (open-angle glaucoma, exfoliative glaucoma and use of antiglaucoma preparations), (2) retinal vascular disease, (3) pregnancy-related maternal complications (mainly antepartum and postpartum hemorrhage) and (4) a set of miscellaneous outcomes, including fertility treatment (procreative management), hydrocele, hypertrophic scar, wrist and hand fracture, migraine and cervical in situ carcinoma (Fig. 7 and Supplementary Table 14).

We considered the results for ASCVD outcomes, dyslipidemia, diabetes, autoimmune disorders and respiratory infections to replicate our previous findings. To provide external validation for the remaining outcomes, we analyzed external datasets (UK Biobank or case-control GWAS studies from disease-specific consortia) with more refined outcome definitions (Supplementary Table 16). In these replications, we confirmed that *IL6* perturbation is associated with lower odds of depression (OR per 24% CRP decrease of 0.993, 95% CI 0.989 to 0.997, *P* = 0.002) and cholelithiasis or cholecystitis (OR of 0.896, 95% CI 0.803 to 0.999, *P* = 0.048). The results across alternative MR methods were consistent, with no evidence of pleiotropy (Supplementary Table 17). Furthermore, we observed a nominally higher risk of migraine (OR of 1.072, 95% CI 1.005 to 1.144, *P* = 0.034), though heterogeneity (*P* = 0.041) and attenuated associations in the weighted median estimator and MR-Egger regression limited interpretability. No significant associations were found for glaucoma, osteoporosis, cervical cancer/dysplasia or cancer of urinary tract organs (Supplementary Table 17).

## Discussion

In this study, we developed a genetic proxy of reduced IL-6 signaling activity that mirrored the effects of pharmacological IL-6 inhibition and showed associations with lower lifetime cardiovascular risk. First, the *IL6* genetic proxy was robustly associated with lower risks of CAD, PAD, atherosclerotic stroke and carotid atherosclerosis across European and East Asian ancestry populations. Second, we found associations with a lower risk of type 2 diabetes, increases in HDL particles and ApoA, and reduced obesity metrics. Third, contrary to concerns about immunosuppression, our analyses showed no evidence of higher infection risk, but revealed lower risks of hospitalization due to pneumonia, critical sepsis and severe COVID-19 infection. Last, phenome-wide analyses supported repurposing potential for depression and gallstone disease, while detecting potential safety signals for migraine, open-angle glaucoma and pregnancy-related maternal hemorrhage.

Our results expand previous evidence on *IL6R* variants^13–20^, demonstrating that genetic perturbation of *IL6* itself is associated with cardiovascular risk. This is relevant as all drugs against IL-6 signaling currently in phase 2 or 3 studies for ASCVD directly target IL-6 rather than IL-6R^10–12^. Several lines of evidence support the involvement of IL-6 in ASCVD. In *Apoe*-deficient mice, injection of IL-6 led to larger atherosclerotic lesions^52^. In population-based studies, circulating IL-6 has been associated with higher risk of incident myocardial infarction, IS and vascular death up to 20 years after the measurement^53^. Post hoc analyses of CANTOS that targeted IL-1β showed reductions in cardiovascular risk that were more profound among patients who achieved reductions in IL-6 levels^26,27^. A previous genetic study of the *IL6* locus was limited by the smaller datasets available at the time, resulting in a selection of variants that did not meet the widely accepted standards for genetic instruments^54^. *IL6* and *IL6R* perturbations may differentially affect the classic, trans-signaling or cluster-signaling pathways^22–25^, thus differentially influencing downstream immune responses. In our study, *IL6* perturbation showed a stronger effect on PAD than *IL6R* perturbation. Interpreting this difference remains challenging owing to limited mechanistic insight into the distinct effects of the two instruments. For example, there are known differences in the immune cell composition of atherosclerotic plaques in femoral arteries, as compared with other vascular beds^55–57^. While our results are consistent, the effect sizes are modest and should be cautiously interpreted, especially within the current pharmacological landscape for ASCVD, where statins, PCSK9 inhibitors and GLP-1 receptor agonists offer large reductions in risk. Still, our findings support associations of *IL6* perturbation with ASCVD across all major vascular beds (coronary, cerebrovascular and peripheral), reinforcing the rationale for advancing IL-6-targeting therapies to clinical testing.

In line with our previous study on *IL6R* perturbation^16^, our findings suggest that IL-6 signaling downregulation via *IL6* may influence metabolic traits. First, we complement existing evidence supporting an involvement of IL6 signaling in the pathophysiology of type 2 diabetes. Case series of patients with rheumatoid arthritis treated with tocilizumab found reductions in HbA1c following IL-6R inhibition^58,59^ and a meta-analysis of prospective studies showed significant associations between higher IL-6 levels and incident type 2 diabetes^60^. However, as we did not observe any association between the *IL6* instrument and HbA1c or other glycemic traits, we suggest cautious interpretation. Neither of the two phase 2 trials testing the anti-IL-6 antibodies ziltivekimab and clazakizumab included glycemic traits as endpoints, despite recruiting substantial numbers of patients with diabetes (71% (ref. ^9^) and 90% (ref. ^11^), respectively). Second, we found genetically downregulated IL-6 signaling via *IL6* to be associated with a favorable lipid profile, including decreases in Lp(a) and increases in HDL particles and ApoA. While earlier trials testing IL-6R blockade reported increases in total cholesterol levels^44,45^, our results align with findings from RESCUE^9^ and POSIBIL_6_ESKD^11^ that assessed lipoprotein fractions. These associations remained consistent when analyzing a panel of 249 metabolomic and lipidomic markers, with largely similar effects for *IL6* or *IL6R* perturbations, suggesting convergence downstream of either IL-6 or IL-6R inhibition. Third, we observed associations of our genetic instrument with lower BMI and WHR, which merit further investigation in future studies.

Our analyses comparing *IL6* versus *IL6R* perturbation offer insights into potentially important safety signals. We found the *IL6* instrument to be associated with a lower risk for severe infections, including pneumonia hospitalization, severe COVID-19 and sepsis. Notably, there was a differential effect between *IL6* and *IL6R* perturbation on hospitalization due to pneumonia. Fatal infections were a major concern related to canakinumab^28^, an anti-IL-1β antibody, and IL-6R inhibitors, such as tocilizumab and sarilumab, are also known to increase infection risk^49,50,61^. While we found that genetic downregulation of IL-6 activity was associated with lower WBC counts, these effects were small and there was no evidence of leukopenia in the PheWAS that tested clinical outcomes. However, given the link between WBC reductions and infection risk, these findings highlight the need to carefully assess clinical infection endpoints in upcoming phase 3 trials. It should be noted that translating genetic associations for acute infection outcomes such as sepsis into clinical or pharmacological insights should be done with caution owing to the conditional nature of infections on environmental exposures. Using population controls without accounting for pathogen exposure may cause outcome misclassification and selection bias, leading to potentially spurious associations^62^. Interestingly, while *IL6R* perturbation has been linked to atopic conditions^16^, *IL6* perturbation showed an inverse association with atopic dermatitis.

Our phenome-wide analyses revealed interesting associations, including lower risks for depression and gallstone disease. The effect on depression is in line with previous analyses of *IL6R* genetic instruments^63,64^, as well as reports on tocilizumab in patients with rheumatoid arthritis^65^. A proof-of-concept randomized trial testing a single intravenous infusion of tocilizumab versus placebo in 30 participants with depression and elevated CRP ≥3 mg l^−1^ (ref. ^66^) found improvements in quality of life, fatigue and cognitive outcomes, but not in the severity of depressive symptoms^67^. While substantial evidence supports a role of inflammation in depression^68,69^, whether IL-6 inhibitors would be a reasonable therapeutic option requires further study. Regarding the effect on gallstone disease, previous evidence is restricted to a case-control study that found higher IL-6 levels among cases compared with population-based controls^70^. Future studies should explore this further, given the limited pharmacological treatments for gallstone disease. On the safety side, we found significant associations with migraine, open-angle glaucoma and maternal pregnancy-related hemorrhage that should be explored further. If confirmed, these findings could have important implications for the use of anti-IL-6 therapies in specific populations, such as patients with migraine and glaucoma or pregnant women.

Our study has limitations. First, the MR paradigm employed here assesses cumulative small lifetime effects of *IL6* genetic variation on clinical outcomes in population-based settings. While our *IL6* instrument explained a low proportion of variance in CRP levels (*R*^2^ = 0.17%), the associations with CRP were robust (*F* statistics >35 for all variants) and the biologically downstream effects were consistent with pharmacological IL-6 inhibition. A very low proportion of explained variance is typical for drug target MR studies focusing on a single genomic locus. For example, instruments of IL-6R, PCSK9 or HMGCR inhibition that have been previously widely used to successfully predict effects of pharmacological interventions all explain <1% of variance in downstream biomarkers^14,16,71^. However, extrapolating these genetic effects that explain up to a 24% difference in CRP levels to predict the effects of clinical interventions, which more aggressively block IL-6 signaling over shorter time frames (for example, an 88% reduction in CRP with ziltivekimab) and in specific patient populations, requires caution. Second, most analyses were limited to European genetic ancestry populations, and caution is warranted when generalizing to other ancestries. Our replication attempts in Biobank Japan faced several limitations including a smaller sample size, reduced number of available variants (6 of 12) and weak instrument strength (only 2 variants with *F* statistics >10). Though these analyses showed directionally consistent associations for the main ASCVD outcomes, the cross-ancestry results remain preliminary and require validation in more diverse populations. Third, a fundamental limitation of all MR analyses is that they assume any genetic associations between the instrument and outcome to occur solely through the exposure and no other pleiotropic pathways. While the consistency of our results across datasets and sensitivity analyses minimizes the probability of such bias, pleiotropic effects cannot be definitely excluded. The Egger intercept was not significant for any of the ASCVD outcomes, suggesting no evidence of directional pleiotropy. In contrast, the significant Egger intercept observed for IL-6 levels suggest that some variants affect IL-6 signaling activity via alternative pathways, not by directly altering circulating IL-6 concentrations. Fourth, the population overlap between the datasets used for genetic association and outcome analyses could introduce weak instrument bias toward the observational estimate (between CRP and outcomes; Supplementary Table 18). However, simulations suggest that relative bias in the case of population overlap is close to the inverse of the mean *F* statistic of the instrument^72^. With a mean *F* statistic of 83 in our study, we estimate any bias due to population overlap to be minimal (<2% in the log-odds scale), thus unlikely to influence the interpretation of our findings. Fifth, we found limited evidence of colocalization between most clinical outcomes and CRP in the *IL6* locus, questioning whether the genetic variants affect the exposure and outcomes via a shared mechanism. However, there was no evidence of distinct causal variants supporting pleiotropy (H3 hypothesis). Instead, for most outcomes, there was evidence of a causal variant only for CRP (H1 hypothesis; Supplementary Table 10). Similarly, the HEIDI test detected no heterogeneity due to linkage of independent causal variants^41^ and SharePro found no evidence of multiple common causal variants^73,74^. This is a common pattern as the threshold to produce non-zero estimates is lower in MR than in colocalization analyses^75^.

In conclusion, we found that genetic variants in *IL6*, which mimic pharmacological IL-6 inhibition, are associated with a lower risk of ASCVD. Our results provide genetic support for the potential of pharmacological therapies directly targeting IL-6 to show meaningful reductions in cardiovascular risk. Our findings further suggest promising repurposing opportunities, including for severe respiratory infections, sepsis, depression and gallstone disease that could be considered for further clinical investigations.

## Methods

### Ethics

The GWASs leveraged for our analyses have received ethical approval by the corresponding institutional review boards of the original studies. The Ethics Committee of the Faculty of Medicine, Ludwig‑Maximilians–Universität München approved the secondary use of these data. Access to the individual-level data from the UK Biobank was granted through an application (number 151281; Principal Investigator: M.K.G.). The UK Biobank obtained approval from the Northwest Multi-Center Research Ethics Committee (number 11/NW/0382). All participants of the UK Biobank have provided written informed consent according to the Declaration of Helsinki.

### Genetic instrument selection

We developed a genetic instrument for the downregulation of IL-6 signaling activity through perturbations in the gene encoding IL-6 (*IL6*). We sought genetic variants within the locus of the *IL6* gene in chromosome 7 that were associated with serum levels of CRP in the largest GWAS of 575,531 European individuals from the Cohorts for Heart and Aging Research in Genomic Epidemiology (CHARGE) Consortium and the UK Biobank^32^. We selected CRP as it is a well-established biomarker of IL-6 signaling activity^14,29,76^ and is used as a readout in clinical trials pharmacologically perturbing IL-6 or the IL-6 receptor^77^. We selected genetic variants associated with CRP levels at a *P* value <5 × 10^−8^ and clumped for LD at *r*^*2*^ < 0.1 according to the 1000 Genomes European reference panel^78^. We selected variants in a region 300 kb upstream or downstream of the *IL6* gene (chr7: 22,466,819 to chr7: 23,071,617 according to the GRCh37/hg19 reference sequence). To avoid confounding effects from neighboring genes, on sensitivity analyses, we restricted the selection to 100 kb and 10 kb upstream or downstream of the *IL6* gene. Owing to the LD structure and our clumping approach, which prioritizes variants with the lowest *P* values, some variants selected in the more restrictive regions may not be retained in the broader set if more highly associated variants in LD are located further from the gene. We calculated the *F* statistic as a proxy of instrument strength and the variance in CRP levels explained for each selected variant (*R*^2^) using formulas applied on summary statistics^79,80^. LocusZoom plots for instruments were generated with the Python package GWASLab v.3.6.6 (ref. ^81^).

### Biological effects of the selected genetic instruments

To examine how the selected variants influence CRP levels, we checked their location in relation to the *IL6* gene (coding or noncoding regions) and, as all variants were in noncoding regions, we explored their effects on *IL6* expression. We tested the effects of the variants on *IL6* expression using the eQTL catalog resource^35^. Among others, the eQTL catalog includes eQTL information for relevant tissues using the Genotype-Tissue Expression (GTEx) v.8 resource (*N* up to 837 donors)^82^ as well as in individual immune cells isolated with single-cell RNA sequencing from peripheral blood mononuclear cells (1M-scBloodNL study, *N* = 120)^83^. We also tested expression in blood in the eQTLGen data (*N* = 31,684; 25,482 whole blood samples and 6,202 peripheral blood mononuclear cell samples)^84^. Furthermore, to test whether the selected instruments influence IL-6 protein levels, we explored the effects of the variants on circulating IL-6 levels, derived from a recent GWAS meta-analysis of 74,679 individuals of European ancestry^36^, as well as on cerebrospinal fluid IL-6 levels (quantified in the context of an aptamer-based proteomics assay), derived from a GWAS analysis of 3,506 individuals of European ancestry^85^.

### Effects of the genetic instruments on IL-6 signaling activity

To test the effects of the genetic instruments on the activity of IL-6 signaling, we next analyzed the effects of the three instruments on CRP levels in individual-level data from the UK Biobank^86^, a prospective cohort study of 502,460 individuals aged 37–73 years recruited between 2006 and 2010. A total of 464,264 participants with available genotyping and CRP data were included in this analysis. The effects of the three instruments were analyzed using GRS constructed with the C + T method^87^. For analyses in the UK Biobank, the weights for all three GRSs were taken from the CHARGE GWAS that did not overlap with UK Biobank^72^. However, this approach might have weakened our instrument as the weights were derived from a less powerful dataset. The reasoning for using the combined CHARGE and UK Biobank dataset for instrument selection was the lack of significant variants for CRP in the *IL6* locus in the isolated CHARGE dataset. Using serum CRP-level data from the baseline assessment in the UK Biobank, the differences in mean and median CRP levels were calculated across fractions of the GRSs (quartiles, deciles, 5th and 2.5th quantiles and percentiles).

### Effects of the genetic instruments on biomarkers influenced by pharmacological IL-6 inhibition

To validate that the developed genetic instruments are proxying pharmacological inhibition of IL-6, we aimed to compare the effects of the genetic instruments with those of the monoclonal antibody ziltivekimab against IL-6 on a set of biomarkers explored as endpoints in the phase 2 randomized trial RESCUE, testing ziltivekimab against placebo in patients with elevated CRP and chronic kidney disease^9^. We analyzed the following readouts on the basis of genetic data availability: fibrinogen, SAA, haptoglobin, Lp(a), low-density lipoprotein cholesterol (LDL-C), HDL-C, ApoB and ApoA. The genetic sources used for this analysis are presented in Supplementary Table 1. The effects of the genetic instruments were compared with the effects of 30 mg ziltivekimab every 4 weeks for 12 weeks versus placebo in the RESCUE trial using Spearman’s correlations^9^.

### Primary cardiovascular outcomes

Our primary outcomes included major ASCVD endpoints, which were analyzed using the largest publicly available GWAS summary statistics datasets. CAD was derived from a meta-analysis encompassing ten de novo studies^88^ (*N* = 1,378,170; 210,842 CAD cases). Myocardial infarction data were sourced from CARDIoGRAMplusC4D^89^. IS was analyzed using GIGASTROKE^90^ (*N* = 1,590,566; 86,668 cases) and its subtypes large artery stroke (9,219 cases), small vessel stroke (13,620 cases), and cardioembolic stroke (12,790 cases), and cryptogenic stroke from SiGN^91^ (*N* = 36,066; 3,593 cases). PAD data came from the Million Veteran Program^92^ (*N* = 243,060; 31,307 cases). Ultrasound-defined carotid subclinical atherosclerosis phenotypes were assessed in CHARGE^93^; the presence of carotid plaque was used as the main readout (N = 48,434), with the mean of maximums of carotid intima media thickness (*N* = 71,128) as an alternative outcome.

### Secondary outcomes and data sources

#### Rheumatological outcomes

Rheumatoid arthritis data included 276,022 individuals (35,871 cases and 240,149 controls)^94^, while polymyalgia rheumatica data comprised 424,651 individuals (8,156 cases and 416,495 controls)^95^ from UK Biobank and FinnGen.

#### Diabetes-related traits and metabolomics

Beyond cardiovascular and rheumatological outcomes, we also explored diabetes-related and metabolic traits. Type 2 diabetes data were sourced from DIAGRAM/DIAMANTE/T2DGGI (*N* = 2,535,601; 428,452 cases)^96^. Glycemic traits (for example, random glucose^97^, HbA1c^98^, fasting insulin^98^, fasting glucose^98^ and 2-h glucose post-challenge^99^) and obesity-related traits (for example, BMI^100^ and WHR^100^) were analyzed using MAGIC (*N* = 281,416–476,326) and GIANT consortia (*N* = 694,649). Visceral adipose tissue (VAT), abdominal subcutaneous adipose tissue (ASAT) and gluteofemoral adipose tissue (GFAT) volumes were derived from UK Biobank MRI data^101^. Circulating metabolites (249 traits) were assessed using NMR profiling from the UK Biobank and Estonian Biobank (*N* = 619,372)^102^.

#### Infectious disease and allergic endpoints

Infectious outcomes included hospital admissions for any infection^103^ *(N* = 700,649; 123,508 cases), pneumonia^103^ (*N* = 700,649; 55,241 cases), urinary tract infection (*N* = 486,484; 21,958 cases)^104^, skin and soft tissue infections (*N* = 405,346; 6,107 cases)^105^, candida infection (*N* = 698,263; 7,286 cases)^103^, sepsis (*N* = 682,462; 18,931 cases)^103^ with its subgroups^104^, and COVID-19 outcomes (*N* = 2,942,817; 159,840 cases)^106^. Data were obtained from UK Biobank, FinnGen and COVID-19-hg GWAS meta-analyses round seven results. Influenza data (*N* = 607,323; 7,712 cases) were sourced from the Million Veteran Program^107^. Allergic endpoints (for example, asthma^108^ and atopic dermatitis^109^) were evaluated in cohorts such as the UK Biobank (*N* = 1,376,071; 121,940 asthma cases).

#### Hematological traits

Hematological traits included RBC, WBC and platelet traits, encompassing a total of 15 specific features. Data were analyzed using the Blood Cell Consortium^110^, with detailed trait descriptions provided in Supplementary Table 1.

### MR analyses

For biomarkers and primary cardiovascular outcomes, we conducted two-sample MR analyses using the fixed-effects IVW approach as the main methodology for the 12-, 8- and 3-variant instruments, whereas other outcomes were analyzed using only the 12-variant instruments^111^. Sensitivity analyses were performed utilizing the random-effects IVW approach, the weighted median estimator, which allows for some pleiotropic variants^112^, as well as MR-Egger regression, which is less powered but generates robust estimates even in the presence of pleiotropy for all variants included in the genetic instrument^113^. Utilizing multiple MR sensitivity analysis approaches and deriving consistent effect size estimates across methodologies that rely on different sets of assumptions provides further support for the validity of the main MR approach, although inherently these approaches may not be as well powered as IVW to detect statistically significant associations^114^. All analyses were scaled to the maximum median difference in CRP levels observed across the GRS distributions in the UK Biobank population to reflect natural genetic variation. To control for multiple comparisons, we applied a Benjamini–Hochberg correction (FDR) to fixed-effects IVW *P* values across the predefined outcome categories (IL-6 signaling biomarkers, trial biomarkers from RESCUE, primary cardiovascular outcomes, glycemic and obesity-related traits, NMR metabolomics, infectious and allergic endpoints, CBC traits and PheWAS). FDR correction was implemented in R4.3.3 using p.adjust (method ‘BH’). Statistical significance was defined as FDR-adjusted *P* < 0.05. To formally assess potential heterogeneity between genetic perturbation of *IL6* and *IL6R*, effect estimates from the present *IL6* instrument were compared with a previously published *IL6R* genetic instrument^15–17,19^ estimates utilizing Cochran’s *Q* test via the R package metafor v.4.6-0 (ref. ^115^). MR analyses were performed using the R package TwoSampleMR v.0.6.7 (ref. ^116^) and MendelianRandomization v.0.10.0 (ref. ^117^).

To complement our two-sample MR analysis and validate findings using individual-level data, we conducted a one-sample MR analysis^118^ in the UK Biobank for CAD, IS and PAD (Supplementary Table 19). We constructed a standardized GRS using the same *IL6* variants, weighted by their CRP effect sizes and standardized (mean of 0, s.d. of 1). Cox proportional hazard models were fitted with age as the time scale (time from birth to event or censoring), using the GRS as the exposure and adjusting for sex (field ID 31), the first ten genetic principal components (PC1–PC10, field ID 22009), genotyping array (field ID 22000) and genetic kinship to other participants (field ID 22021) to account for population structure and potential relatedness. Linear regression modeled the association between GRS and log-transformed CRP levels, adjusting for the same covariates and baseline age (field ID 21022). The MR estimate was obtained by dividing the log-hazard coefficient from the Cox model by the regression coefficient from the CRP model. Standard errors were calculated using the delta method, from which 95% CIs were derived assuming normality.

### Replication of the cardiovascular effects in an East Asian population

As the datasets used in our analyses were largely from European-ancestry individuals, we then performed a cross-ancestry replication of our results in an East Asian population. Using data from Biobank Japan, we searched for the variants we included in our instruments and then reclumped for LD based on an East Asian LD reference panel. We used weights from summary GWAS statistics for serum CRP from 75,391 individuals^42^, and then tested associations with CAD (29,319 cases and 183,134 controls), PAD (3,593 cases and 208,860 controls) and IS (17,671 cases and 192,383 controls)^43^.

### Colocalization analyses

To examine the colocalization of putative causal variants between IL-6 and traits of interest, we used coloc v.5.2.3. The analysis was performed with default parameters and prior probabilities (*P*_1_ = *P*_2_ = 10^−4^, *P*_12_ = 10^−5^) focusing on variants located within 300 kb upstream and downstream of the IL-6 locus. We considered a PP.H4 value (the probability of a shared causal variant between two traits) greater than 0.80 as evidence of colocalization and a PP.H4 greater than 0.50 as indicative of colocalization. Additionally, we conducted colocalization analysis using SharePro^73^, assuming multiple causal variants. We utilized default parameters: a maximum of 10 effect groups and a prior colocalization probability of 1 × 10^−5^. To further assess heterogeneity due to LD, we conducted the HEIDI test using *SMR* v.1.3.1 (ref. ^41^). The HEIDI test evaluated homogeneity in the effects of *IL6* variants on clinical outcomes. Analyses used a minor allele frequency threshold of 0.01, with the 1000 Genomes European reference panel. A HEIDI *P* value <0.05 indicated significant heterogeneity due to LD.

### PheWAS study

We conducted a phenome-wide MR analysis to systematically assess the associations between genetically downregulated IL-6 signaling and a broad spectrum of clinical outcomes using data from the FinnGen study, a population-based cohort comprising 500,348 individuals of Finnish ancestry. The current analysis was based on the latest R12 data release^51^. From the initial set of outcomes, we restricted the analysis to 1,285 binary traits with at least 1,000 cases, ensuring sufficient statistical power to detect robust associations. Similar to the other outcomes, the primary method was the fixed-effects IVW MR. To identify statistically significant results, we applied multiple testing correction using the FDR approach, with a significance threshold of FDR-corrected *P* value <0.05. For further validation, we conducted a series of sensitivity analyses to assess the robustness of the results and detect potential violations of MR assumptions. Specifically, associations were considered significant only if they demonstrated (1) consistent effect directionality with *P* < 0.05 in the weighted median estimator, (2) no evidence of horizontal pleiotropy, as indicated by a nonsignificant MR-Egger intercept (*P* > 0.05) and (3) in the presence of heterogeneity (Cochran’s *Q* statistic *P* < 0.05), concordant effect directionality across weighted median and MR-Egger analyses, with the MR-Egger effect size deviating by no more than 20% from the IVW estimate. This rigorous filtering strategy ensured that only robust associations meeting stringent statistical criteria were retained, minimizing the risk of spurious findings. To further validate the robustness of the significant associations identified in FinnGen, where available we performed replication analyses using independent, large-scale external datasets (Supplementary Table 16).

### Reporting summary

Further information on research design is available in the Nature Portfolio Reporting Summary linked to this article.

## Supplementary information

Reporting Summary

Supplementary Tables 1–19

## Source data

Source Data Figs. 2, 3, 5 and 6 and Extended Data Figs 2 and 3 Statistical source data.

## Data Availability

All data used in this study comprise summary-level GWAS statistics from established consortia (publicly available or available upon request) and individual-level records from UK Biobank (application number 151281). The datasets can be accessed as follows: PAD GWAS data are available on request via dbGaP (accession: phs001672.v12.p1), COVID-19 GWAS data were obtained from the COVID-19 Host Genetics Initiative (https://www.covid19hg.org/results/r7/), type 2 diabetes data were sourced from DIAGRAM/DIAMANTE/T2DGGI (https://www.diagram-consortium.org/downloads.html), glycemic traits data were obtained from the MAGIC consortium (http://magicinvestigators.org/), BMI and WHR data were sourced from the GIANT consortium (https://portals.broadinstitute.org/collaboration/giant/index.php/GIANT_consortium), blood cell count data were obtained from the Blood Cell Consortium (http://www.mhi-humangenetics.org/en/resources/) and PheWAS MR analyses utilized data from FinnGen R12 (https://r12.finngen.fi/). Summary statistics for alcohol dependence and psychiatric traits, including major depressive disorder and depression, were sourced from the Psychiatric Genomics Consortium (https://pgc.unc.edu/for-researchers/download-results/). Additional datasets were accessed through the GWAS Catalog (https://www.ebi.ac.uk/gwas/). Detailed references for each dataset are provided within the paper and supplementary materials.

## Code availability

All analyses were performed using R (v.4.3.3), Python (v.3.9.1) and PLINK (v.2.00a3.3LM), as detailed in Methods. No novel computational methods or custom codes were developed that were essential to the paper’s conclusions. All analytical scripts are openly available via GitHub at https://github.com/DeepVasc-Lab/IL6-genetic-perturbation.
