# Supplementary material for: IL6 genetic perturbation mimicking IL-6 inhibition is associated with lower cardiometabolic risk
Source: Nat Cardiovasc Res. 2025 Aug 26;4(9):1172–86. doi: 10.1038/s44161-025-00700-7 (PMC12436178; doi:10.1038/s44161-025-00700-7)
Supplement: Supplementary file 1 — Reporting Summary [file 44161_2025_700_MOESM1_ESM.pdf]

nature portfolio

Corresponding author(s): Marios K. Georgakis

Last updated by author(s): Aug 12, 2025

Reporting Summary

Nature Portfolio wishes to improve the reproducibility of the work that we publish. This form provides structure for consistency and transparency in reporting. For further information on Nature Portfolio policies, see our Editorial Policies and the Editorial Policy Checklist.

Statistics

For all statistical analyses, confirm that the following items are present in the figure legend, table legend, main text, or Methods section.

n/a	Confirmed	
☐	☒	The exact sample size (*n*) for each experimental group/condition, given as a discrete number and unit of measurement
☐	☒	A statement on whether measurements were taken from distinct samples or whether the same sample was measured repeatedly
☐	☒	The statistical test(s) used AND whether they are one- or two-sided<br>*Only common tests should be described solely by name; describe more complex techniques in the Methods section.*
☐	☒	A description of all covariates tested
☐	☒	A description of any assumptions or corrections, such as tests of normality and adjustment for multiple comparisons
☐	☒	A full description of the statistical parameters including central tendency (e.g. means) or other basic estimates (e.g. regression coefficient) AND variation (e.g. standard deviation) or associated estimates of uncertainty (e.g. confidence intervals)
☐	☒	For null hypothesis testing, the test statistic (e.g. *F*, *t*, *r*) with confidence intervals, effect sizes, degrees of freedom and *P* value noted<br>*Give P values as exact values whenever suitable.*
☒	☐	For Bayesian analysis, information on the choice of priors and Markov chain Monte Carlo settings
☒	☐	For hierarchical and complex designs, identification of the appropriate level for tests and full reporting of outcomes
☐	☒	Estimates of effect sizes (e.g. Cohen's *d*, Pearson's *r*), indicating how they were calculated

Our web collection on statistics for biologists contains articles on many of the points above.

Software and code

Policy information about availability of computer code

Data collection	No software was used for data collection.
Data analysis	Analyses were conducted using R (v4.3.3), Python (v3.9.1), and PLINK (v2.00a3.3LM). Mendelian randomization was performed using TwoSampleMR (v0.6.7) and MendelianRandomization (v0.10.0) packages. SMR (v1.4.0) was used for HEIDI heterogeneity testing, and coloc (v5.2.3) for colocalization analysis.<br><br>No novel computational methods or custom codes were developed that were essential to the manuscript's conclusions. All analytical scripts are openly available at (https://github.com/DeepVasc-Lab/IL6-genetic-perturbation).

For manuscripts utilizing custom algorithms or software that are central to the research but not yet described in published literature, software must be made available to editors and reviewers. We strongly encourage code deposition in a community repository (e.g. GitHub). See the Nature Portfolio guidelines for submitting code & software for further information.

Data

Policy information about availability of data

All manuscripts must include a data availability statement. This statement should provide the following information, where applicable:

- Accession codes, unique identifiers, or web links for publicly available datasets
- A description of any restrictions on data availability
- For clinical datasets or third party data, please ensure that the statement adheres to our policy

All data used in this study comprise summary statistics-level GWAS statistics from established consortia (publicly available or available upon request) and individual-level records from UK Biobank (application no. 151281). The datasets can be accessed as follows: PAD GWAS data are available from dbGaP (accession: phs001672.v12.p1); COVID-19 GWAS data were obtained from the COVID-19 Host Genetics Initiative (https://www.covid19hg.org/results/r7/), type 2 diabetes data were sourced from DIAGRAM/DIAMANTE/T2DGGI (https://www.diagram-consortium.org/downloads.html), glycemic traits data were obtained from the MAGIC consortium (http://magicinvestigators.org/downloads/), BMI and WHR data were sourced from the GIANT consortium (https://portals.broadinstitute.org/collaboration/giant/index.php/GIANT_consortium), and blood cell count data were obtained from the BCX (http://www.mhi-humangenetics.org/en/resources/), and PheWAS MR analyses utilized data from FinnGen R12 (https://r12.finngen.fi/).Summary statistics for alcohol dependence and psychiatric traits, including major depressive disorder and depression, were sourced from the Psychiatric Genomics Consortium (PGC) (https://pgc.unc.edu/for-researchers/download-results/). Additional datasets were accessed through the GWAS Catalog (https://www.ebi.ac.uk/gwas/). Detailed references for each dataset are provided within the manuscript and supplementary materials.

Research involving human participants, their data, or biological material

Policy information about studies with human participants or human data. See also policy information about sex, gender (identity/presentation), and sexual orientation and race, ethnicity and racism.

Reporting on sex and gender	Not applicable. We used the public data, and the participants were the same as the original data and vary from dataset to dataset. Detailed sample information can be accessed in the references.
Reporting on race, ethnicity, or other socially relevant groupings	Yes, there are descriptions of genetic ancestry in supplementary tables. We used the public data, and the participants were the same as the original data and vary from dataset to dataset.
Population characteristics	The population characteristics for UK Biobank used in this study are listed in Supplementary Table 3.
Recruitment	Not applicable
Ethics oversight	The GWASs leveraged for our analyses have received ethical approval by the corresponding institutional review boards of the original studies.

Note that full information on the approval of the study protocol must also be provided in the manuscript.

Field-specific reporting

Please select the one below that is the best fit for your research. If you are not sure, read the appropriate sections before making your selection.

☒ Life sciences ☐ Behavioural & social sciences ☐ Ecological, evolutionary & environmental sciences

For a reference copy of the document with all sections, see nature.com/documents/nr-reporting-summary-flat.pdf

Life sciences study design

All studies must disclose on these points even when the disclosure is negative.

Sample size	The sample sizes for each GWAS summary statistic are provided in the supplementary tables. We selected the largest and most recent datasets available to maximize statistical power, with sample sizes determined by the original contributing studies. The adequacy of sample sizes was evaluated through instrument strength assessment (F-statistics) and power calculations to ensure sufficient power for detecting clinically meaningful effects in Mendelian randomization analyses.
Data exclusions	No data were excluded arbitrarily. All exclusions were based on predefined criteria, such as removing variants with insufficient statistical significance ($p > 5e-8$) or linkage disequilibrium (LD, $r^2 > 0.1$), as described in the Methods section.
Replication	Replication was conducted across multiple datasets and populations. Cardiovascular and metabolic outcomes were validated in both European and East Asian populations. Additionally, partial validation of some PheWAS-MR findings was achieved in independent datasets.
Randomization	Samples were not randomized, as this is a Mendelian randomization study utilizing naturally occurring genetic variation.
Blinding	Blinding was not applicable as the analyses were based on publicly available summary statistics, not individual-level data or experimental interventions.

Reporting for specific materials, systems and methods

We require information from authors about some types of materials, experimental systems and methods used in many studies. Here, indicate whether each material, system or method listed is relevant to your study. If you are not sure if a list item applies to your research, read the appropriate section before selecting a response.

Materials & experimental systems

n/a	Involved in the study
☒	☐ Antibodies
☒	☐ Eukaryotic cell lines
☒	☐ Palaeontology and archaeology
☒	☐ Animals and other organisms
☒	☐ Clinical data
☒	☐ Dual use research of concern
☒	☐ Plants

Methods

n/a	Involved in the study
☒	☐ ChIP-seq
☒	☐ Flow cytometry
☒	☐ MRI-based neuroimaging

Plants

Seed stocks	Not applicable
Novel plant genotypes	Not applicable
Authentication	Not applicable
